# Foreign Bodies in the Rectum

**Published:** 1911-11

**Authors:** 


### Foreign Bodies in the Rectum

Dr. T. L. Hazzard of Pittsburg, at the Los Angeles meeting of the American Proctologic Society, reported the following cases:

1. Girl, two years old, nearly the whole of match fixed in rectum.

2. Boy, slightly older, bone of frog's leg similarly placed.

3. Adult male, 3-ounce Baltimore oval bottle, introduced from below; no questions asked as the patient would have lied.

4. Male aged 45; thin tumbler 2 inches in diameter at top, 3½ high. (In regard to the first two cases, we may call attention to the fact, realized by few of the laity or even of the profession who have not used stool sieves considerably, that a large amount of foreign material is habitually ingested. We have obtained several chicken ribs from the passages of a supposed nursling; have seen large splinters of wood stuck in the rectum of persons who considered themselves careful eaters and who denied the possibility of introduction from below; while the frequency of finding large seeds, bone fragments, large masses of vegetable fibre and skin, labels, etc., renders it difficult to draw the line between indigestible edible residue and foreign bodies. Years ago, in the museum of Harvard, we wonderd at the carelessness of the attendants in leaving a label without a specimen, and at the parsimony of so rich an institution in using a large catsup bottle as a container, until it gradually dawned upon us that the bottle itself was the specimen.—Editor)

Bubonic Plague.—J. Campbell MacDiarmid and J. Howard Lawry, *New Zealand Medical Journal,* August, 1911, report seven cases of plague at the Auckland Hospital. Five were bubonic, all recovering. Two were pneumonic, one recovering, one dying. The non-fatal case occurred in the nurse who took care of the other and was apparently primarily a respiratory infection. However, the first manifestation was an acute nephritis, the bacilli not being found in the sputum till the seventh day. The treatment was mainly symptomatic, strychnine or quinine being used as a stimulant, the buboes being treated by hot and cold applications in different cases, preference being given to 10 per cent. aqueous solution of ichthyol, with frequent change of dressings, and hydrogen peroxid for sloughs. The average stay in the hospital was forty-nine days.

---

THE September number of *"Surgery, Gynecology and Obstetrics,"* contains a symposium on the relative merits of suprapubic and perineal prostatectomy, composed of papers read before The Urological Society, by Hugh Young, Bentley Squier, William Belfield and Parker Syms. Squier defends the suprapubic method and describes an original technic in which the bladder is opened as high up as possible and the gland enucleated through an opening in the roof of the prostatic urethra. He also demonstrates that the external sphincter surrounds the intravesical mass, photographs showing the groove upon the enucleated organ caused by its compression. The technic of enucleation is illustrated by excellent diagrams.

Young describes his well known technic and mentions the use of his median bar punch as well as quoting statistics of several hundred operations.

Belfield emphasizes the point that the urethra should be opened just behind the external sphincter. Syms insists that the finger must be swept around the internal sphincter to be sure that there are no intravesical projections. All of the papers emphasize the necessity of careful preparatory treatment.

---

NICKEL SULPHATE.—Louis Kolipinski of Washington, *Monthly Cyclopaedia and Medical Bulletin,* June 1911, states that the first medicinal use of nickel was by J. Y. Simpson in 1853, as a general tonic. Nickel does not seem to be toxic as a base—negative results from use of cooking utensils, feeding experiments, etc.—but its soluble salts are irritant, producing nausea, vomiting, diarrhoea, giddiness, pulse depression in a dose of 1-3 gram or thereabouts. The average adult dose of the sulphate is about 1 grain, various authorities having established a safe range of 3—10 or 15 centigrams. The therapeutic value of nickel may be summed up as a safe substitute for arsenic—a general tonic and nervine, hence useful in migraine, urticaria, chronic psoriasis, chorea, stammering, tic douleureux, masturbation and various other neurotic sexual disturbances.

Pellagra.—Dr. George C. Mizell of Atlanta, *Journal-Record of Medicine,* September, 1911, continues his contention that pellagra is due to the excessive use of cotton seed and other non-drying oils. We must not overlook the fact that cotton seed oil is a valuable nutrient. At the same time, it is possible that Mizell has hit upon the true explanation of pellagra or has at least afforded a hint worth careful investigation. In this, as in other dietetic problems, it must be remembered that the proper balancing of a ration, is of the utmost importance. His article, at any rate, contains a great deal of interesting statistic matter regarding the prevalence of pellagra, the kinds of non-drying oils available in different parts of the world and the economic uses of cotton seed and its derivatives.

---

The Relation of Vaccine Therapy to the Treatment of Certain Diseases of the Skin, D. King Smith. (*Jour. Cutan. Dis.,* Aug. 1911).

Acne cases are divided into three classes:

A. Those due to the Acne bacillus.

B. To Acne bacillus and Staphylococcus Albus.

C. To Staphylococcus Albus.

In Class "A"—cases with comedones, papules and papulo-pustules—a vaccine of the acne bacillus was used; dose 5 millions twice weekly. Results were unsatisfactory but in connection with general and local treatment, Dr. Smith considers the vaccine useful.

In Class "B,"—cases with papulo-pustules and pustules—a mixed virus, of 5 millions of Acne bacilli and 10 millions of the Albus, was employed with fifty per cent. of success.

In Class "C,"—cases with deep-seated pustules and much scarring—the Staphylococcus Albus in dose of 10 millions was given with excellent results.

In Furunculosis a vaccine composed of the Staphylococcus Aureus, Albus and Citreus, dose 125 millions twice weekly for three or four weeks gave good results in ninety per cent. of the cases. Similar results were obtained in carbuncles.

In Coccogenic Sycosis, 125 millions of the Albus, twice a week, produced good results, notably in acute cases.

In Erysipelas brilliant results were obtained by the use of a composite stock virus of devitalized streptococci derived from several different strains, with a first dose of 250 millions in severe cases and 5 millions if less severe. If there are signs of improvement, the dose is repeated the following day, the general rule being: "The more severe the case and the less satisfactory the clinical response, the smaller the dose."

In Eczema of the squamous type excellent results were obtained from the Albus vaccine.

A means of controlling the hemorrhage from inoperable Neoplasms of the Bladder: With Cases Illustrating the Trypsin and Hodenpyl-Serum Therapy.—L. Bolton Bangs, New York, states that one can relieve the hemorrhage from tumors of the bladder, which are a cause of great apprehension to the patient, by the use of a solution of creolin injected into the bladder. Creolin is an antiseptic with hemostatic properties. The author gives the histories of four cases in which it was used with benefit. A 1 per cent. solution is found to be efficient for this purpose. It relieves irritability of the bladder at the same time that it reduces the loss of blood, and in some cases it reduces the size of the tumor. The author has found no value in the use of ascitic serum or of trypsin in cases of cancer of the bladder. In one case the Hodenpyl serum was used persistently for a long period without any apparent benefit. Although there is no permanent cure to be hoped for by the use of the creolin treatment, still the life of the patient is made more comfortable and often prolonged considerably.—*Medical Record,* August 19, 1911.

Paralysis of the Upper Extremity Due to Nerve Injuries.—Nathan Jacobson, Syracuse, N. Y., presents the histories of several cases of nerve injuries of the upper extremity with remarks on the diagnosis and treatment. The most frequent cause of partial paralysis of the upper extremity is injury of the musculo-spiral nerve; this occurs especially in fractures of the humerus, the nerve being torn at the time of the accident, or becoming compressed, or adherent to the callus in the process of repair. The nerve can also be injured at a lower point, in the lower third of the arm in fractures. The injury is not always recognized at the time of the fracture but appears when the splints are removed, with the characteristic wrist-drop and inability to extend the fingers. In two of the cases presented the nerve was adherent to the callus and separation of the nerve from the cicatrix brought about entire recovery. When there are visible changes in the nerve as a result of injury recovery is slow. A case of fracture of the neck of the scapula with displacement of the head of the bone and adhesions to the brachial plexus caused complete paralysis of sensation and motion. No apparent injury of the nerve occurred and a few days after operation motion began to return and recovery ensued. When there is complete division of the nerve trunk we have a different condition entirely. In such a case after uniting the nerve-ends six months after the injury good results were obtained; sensation was completely restored, and many muscular movements have returned. If a nerve is reunited soon after injury the immediate results are not better than after remote operation. If suppuration has occurred in the wound with granulation one will not get restoration of function without operation.—*Medical Record, August* 19, 1911.

---

RECOLLECTIONS OF CHOLERA EPIDEMICS IN RUSSIA.—G. A. Friedman, New York, presents recollections of his service as sanitary physician in the cholera barracks near St. Petersburg in 1894. A novice in this work, the author recognized that there were numerous cases of ordinary diarrhea, and soon the epidemic began. The occurrence of many cases of diarrhea in any place exposed to cholera should lead a physician to suspect the beginning of an epidemic. Persons suffering from gastrointestinal disorders are predisposed to cholera. Mouth infection is the most important source of the disease. Cholera appears frequently among priests after funerals in Russia, where custom causes them to partake of food and drink in the infected house after the funeral. Alcohol, a fasting stomach, and choleraphobia are predisposing causes. Bacteriological examinations are important early in the disease. Later the picture is striking; profuse diarrhea, rice-water stools, vomiting, muscular cramps, hoarse voice, and anuria are present. The author found hot baths of use in the algid stage. Salol, followed by naphthalin and benzo-naphthol, was used to control diarrhea. Opium is not to be used.—*Medical Record,* August 19, 1911.

---

CHEMICAL AND BIOLOGICAL AIDS IN THE EARLY DIAGNOSIS OF GASTRIC CANCER.—A. E. Austin, Boston, Mass., states that when patients are operated on before there is extensive stasis it is found that free hydrochloric acid is present in many cases. Positive hemolysis was found in 22 out of 26 cases of gastric cancer, while it was present but once in 60 noncarcinomatous cases of gastric trouble. When pepsin is found in marked amounts in the urine and there is little or none in gastric contents this points to gastric cancer. The presence of tryptophan in gastric contents is an indication of gastric cancer. The bromine test of stomach contents will give a violet color in the presence of tryptophan. In a series of cases tested by the author this test was negative in 20 cases out of 22, and one of these showed a palpable tumor. The tryptophan test is considered by the author the most available test for cancer that can be used in a clinic.—*Medical Record,* August 19, 1911.

